# Treatment of Early-Stage Gynecological Cancer-Related Lower Limb Lymphedema by Lymphaticovenular Anastomosis—The Triple Incision Approach

**DOI:** 10.3390/medicina58050631

**Published:** 2022-05-01

**Authors:** Anna Amelia Caretto, Gianluigi Stefanizzi, Giorgia Garganese, Simona Maria Fragomeni, Alex Federico, Luca Tagliaferri, Bruno Fionda, Alessandro Cina, Giovanni Scambia, Stefano Gentileschi

**Affiliations:** 1 Facoltà di Medicina e Chirurgia, Università Cattolica del Sacro Cuore, 00168 Rome, Italy; annaamelia.caretto01@icatt.it (A.A.C.); giorgia.garganese@policlinicogemelli.it (G.G.); stefano.gentileschi@unicatt.it (G.S.); 2 Dipartimento Scienze della Salute della Donna, del Bambino e di Sanità Pubblica, Fondazione Policlinico Universitario A. Gemelli IRCCS, 00168 Rome, Italy; gianluigi.stefanizzi@policlinicogemelli.it (G.S.); simona.fragomeni@policlinicogemelli.it (S.M.F.); alex.federico@policlinicogemelli.it (A.F.); 3 Mater Olbia Hospital, Gynecology and Breast Care Center, 07026 Olbia, Italy; 4 Dipartimento di Diagnostica per Immagini, Radioterapia Oncologica ed Ematologia, Fondazione Policlinico Universitario A. Gemelli IRCCS, 00168 Rome, Italy; luca.tagliaferri@policlinicogemelli.it (L.T.); bruno.fionda@policlinicogemelli.it (B.F.); alessandro.cina@policlinicogemelli.it (A.C.)

**Keywords:** gynecological cancer, endometrial cancer, cervical cancer, lymphedema, pelvic lymphadenectomy, supermicrosurgery, lymphaticovenular anastomosis, lymphedema treatment, personalized medicine, quality of life

## Abstract

*Background and Objectives*: Lower extremity lymphedema (LEL) is one of the most relevant chronic and disabling sequelae after gynecological cancer therapy involving pelvic lymphadenectomy (PL). Supermicrosurgical lymphaticovenular anastomosis (LVA) is a safe and effective procedure to treat LEL, particularly indicated in early-stage cases when conservative therapies are insufficient to control the swelling. Usually, preoperative assessment of these patients shows patent and peristaltic lymphatic vessels that can be mapped throughout the limb to plan the sites of skin incision to perform LVA. The aim of this study is to report the efficacy of our approach based on planning LVA in three areas of the lower limb in improving early-stage gynecological cancer-related lymphedema (GCRL) secondary to PL. *Materials and Methods*: We retrospectively reviewed the data of patients who underwent LVA for the treatment of early-stage GCRL following PL. Patients who had undergone groin dissection were excluded. Our preoperative study based on indocyanine green lymphography (ICG-L) and color doppler ultrasound (CDU) planned three incision sites located in the groin, in the medial surface of the distal third of the thigh, and in the upper half of the leg, to perform LVA. The primary outcome measure was the variation of the mean circumference of the limb after surgery. The changes between preoperative and postoperative limbs’ measures were analyzed by Student’s *t*-test. *p* values < 0.05 were considered significant. *Results*: Thirty-three patients were included. In every patient, three incision sites were employed to perform LVA. A total of 119 LVA were established, with an average of 3.6 for each patient. The mean circumference of the operated limb showed a significant reduction after surgery, decreasing from 37 cm ± 4.1 cm to 36.1 cm ± 4.4 (*p* < 0.01). *Conclusions*: Our results suggest that in patients affected by early-stage GCRL secondary to PL, the placement of incision sites in all the anatomical subunits of the lower limb is one of the key factors in achieving good results after LVA.

## 1. Introduction

Pelvic lymphadenectomy (PL) is frequently performed in patients affected by gynecological malignancies, and lower extremity lymphedema (LEL) is one of its most relevant chronic and disabling sequelae. LEL incidence after gynecological cancer therapy involving PL is estimated to be very high, namely between 20% and 40% [1]. Many studies have clearly shown a further increase in this risk in the presence of overweight patients, in case of the need to remove the most distal external iliac lymph nodes (so-called CINDEIN), or when surgery is associated with radiation therapy [2,3,4,5,6].

Intrapelvic lymph node dissection creates a blockage of lymph outflow from the inguinal basin with increased pressure and lymph stasis, leading to progressive sclerotic degeneration of the groin lymph nodes that lose both lymphatic pump and immune function [7,8]. The lymph stasis causes an initial increase in endoluminal pressure with ectasia of the lymphatic vessels (ectasis type), followed by wall thickening with lumen reduction (contraction type) and progressive degeneration and sclerosis with loss of peristaltic function (sclerosis type) [9,10]. This process starts in the groin and usually progresses from proximal to distal in the lower limb, triggering lymphedema and leading to interstitial lymphatic fluid collection, chronic inflammation, adipose deposition, hyperkeratosis, and fibrosis. 

The quality of life of these patients is greatly affected by the significant physical and psychological impact, reduction in daily activities, and economic burden [11,12,13,14]. Surgical treatments are indicated when conservative therapies are ineffective in controlling the swelling or for patients with early-stage disease with the goal to reduce or eliminate the use of compressive garments over time [15]. For these reasons, early diagnosis and precise evaluation are crucial for lymphedema management, reducing the risk of progression of lymphatic damage and the development of complications [14], such as soft tissue infections, that further worsen the symptoms [15]. 

Lymphoscintigraphy is the first examination employed to confirm the diagnosis of lymphedema of the extremities. It allows for assessing the lymphatic pathways, calculating transport index, estimating dermal backflow (DB), and evaluating any indication for surgery. Indocyanine green lymphography (ICG-L), first introduced by Unno et al. [16], is a non-invasive and useful method that assesses lymphatic function in real-time, allowing surgeons to map lymphatic vessels preoperatively and to plan surgical incisions for lymphaticovenular anastomosis (LVA) [17]. As the disease worsens, ICG-L patterns of fluorescence progress from linear to splash, stardust, and diffuse [18,19,20].

In patients affected by early-stage LEL secondary to PL, lymphoscintigraphy often shows ectasic lymphatic vessels with compensatory pathways and functioning lymph nodes in the groin that uptake the radiotracer. Similarly, the early phase of ICG-L is mainly characterized by linear or splash patterns with ectasic and compensatory vessels and by stardust or diffuse pattern of DB in the late phase [20]. Many surgeons agree that LVA is particularly indicated in these cases because lymphatic vessels are still patent and peristaltic to pump lymph into the venous system [11,12,14,21,22,23,24].

Otherwise, there is no consensus about the placement of skin incisions for LVA throughout the limb. The incision sites are usually decided according to ICG-L findings and the surgeon’s preference.

In patients affected by early-stage LEL following PL, our approach is to perform LVA in three sites, according to lymphoscintigraphy and ICG-L findings. The first site in the upper part of the leg, the second in the inner surface of the thigh at the level of the upper edge of the patella, the so-called Seki point [25,26], and the third in the groin. We developed this approach aiming to treat the swelling of every anatomical subunit of the lower limb. This study reports the efficacy of this “three incisions method” for the treatment of early-stage LEL secondary to PL performed for gynecological cancer. 

## 2. Materials and Methods

We performed a retrospective observational study reviewing the data collected in the clinical records and during the follow-up of all patients affected by early-stage LEL secondary to PL performed for gynecological cancer who underwent LVA between 2015 and 2021. Patients who had undergone groin dissection were excluded. 

We planned LVA in three incision sites in patients showing swelling of the whole limb from the groin to the ankle, partially or completely refractory to conservative therapy. This evaluation was performed with the physical therapist at least 6 months after conservative treatment optimization.

**Figure 1:**
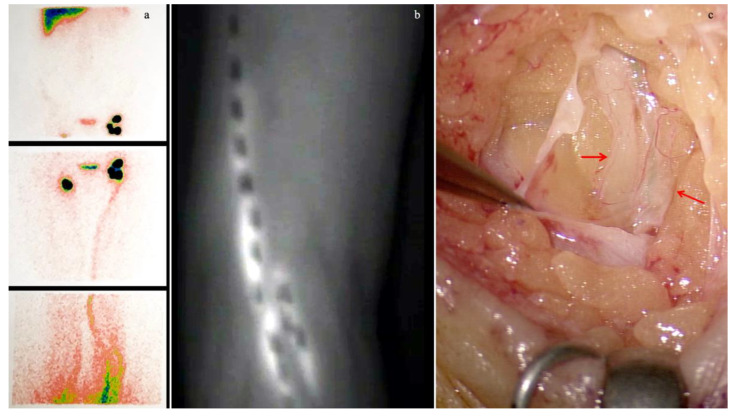
This figure shows the instrumental and intra-operative features typical of early-stage lower limb lymphedema. (**a**) Preoperative lymphoscintigraphy aimed at the superficial lymphatic system of a patient affected by early-stage lymphedema of the left lower limb secondary to Pelvic Lymphadenectomy (PL) for gynecological cancer. The lower part of the picture shows the leg functioning ectasic pathways with some extravasation in the left leg. In the middle figure, functioning vessels are present in the thigh. The image also reveals functioning inguinal lymph nodes that uptake the radiotracer. In the upper part of the picture, no pelvic lymph nodes are present; (**b**) Indocyanine Green Lymphography (ICG-L) of the same patient shows splash pattern, typical of ectasic lymphatic vessels; (**c**) intra-operative picture of the same patient during surgery shows ectasic lymphatic vessels, indicated by red arrows. In one of the two vessels, green dye is visible inside the lumen.

We considered “early-stage” patients classified as stage I-II, according to the International Society of Lymphology (ISL) [27], showing a good visualization of the superficial lymphatic circulation and of the inguinal lymph nodes at preoperative lymphoscintigraphy and patent lymphatic vessels with predominant splash pattern at the early phase of ICG-L (Figure 1).

The primary outcome measure of this investigation was the variation of the mean circumference of the limb after surgery. The variables of interest that we collected were the patients’ age, height, weight, BMI, smoking habits, comorbidities, previous surgery, cancer site, number of lymph nodes removed during lymphadenectomy, months of lymphedema duration, lymphoscintigraphy and ICG-L findings, intra-operative features of lymphatic vessels, recipient venules and anastomosis, months of follow-up, previous soft-tissue infections (in terms of lymphangitis, erysipelas, cellulitis) previous radiation therapy, girth measurements every 4 cm on the limb starting from the ankle joint, before surgery and during the follow-up, and the class of compression of the elastic garment needed to maintain good decongestion of the limb. To reduce the possibility of errors in the detection and registration of the data, we performed a double check, whenever possible, by comparing the data present in the digital clinical archive of our institution, which are available for outpatient and hospitalized patients, and in Redcap, a platform available in our hospital for online databases and surveys.

### 2.1. Preoperative Planning and Surgical Technique

To perform the procedure with the greatest possible limb decongestion, every patient was prepared using bandages for 7 days before surgery. The day before surgery, the patients were assessed by ICG-L and color doppler ultrasound (CDU), establishing lymphatic and venular mapping of the limb to plan the sites of skin incisions. In every patient, three areas were carefully examined to plan the skin incisions for LVA: the leg, the distal third of the thigh, and the groin. 

ICG-L was accomplished according to a method slightly different from the standard one that we routinely employ for the preoperative study of lymphedema and lymphoceles widely reported in the literature [8,18,20,28,29]. A total of 0.2 mL of ICG (Verdye, Diagnostic Green LLC, Farmington Hills, MI, United States) was injected intradermally at the first web space of the foot and at the lateral surface of the Achilles tendon in the affected limb. Immediately after, 5 more boluses of 0.2 mL of ICG were intradermally and subcutaneously injected at the level of the knee in the medial and anterior skin of the limb, starting from the medial aspect, every 5 cm following a horizontal line. Then quickly, 5 more injections of 0.2 mL of ICG were performed approximately at the level of the middle of the thigh, in the medial and anterior surface of the limb, every 5 cm, starting from the medial aspect and following a horizontal line. After ICG injection, dynamic fluorescence images were obtained by an infrared camera system (Fluobeam-Fluoptics, Fluoptics Europe 44 rue des Berges, 38000 Grenoble – France). Both still images and videos were recorded. The lymphatic vessels showing a linear or splash pattern were marked on the skin with dotted lines [30]. After marking the linear course of the lymphatic vessels on the skin, we searched for the points in which a linear course of lymphatic vessels entered an extravasation area of diffuse DB pattern. We assumed that these points are sites of lymphatic flow immediately upstream to points of obstruction of the lymphatic circulation. These areas have been recently called “overlapping” areas, by Yamamoto et al. [31]. In these regions, dilated lymphatic vessels can often be found. If diffuse or stardust areas were not present in the early phase of ICG-L, we searched for the areas that showed a linear or splash pattern during the early phase and DB pattern in the plateau phase. These regions were circled with a green skin marker to be investigated after the completion of ICG-L by CDU. The leg was examined and marked first. Then, the inner surface of the distal third of the thigh was explored in the same way. If linear or splash patterns of fluorescence were visible at this level, the lymphatic vessels were marked similarly to the leg; however, in 11 patients, probably due to the deeper course of lymphatic vessels, linear or splash patterns were not detectable in the distal third of the thigh. In these cases, we investigated only by CDU after the completion of ICG-L, the inner surface of the thigh at the upper edge of the patella in the point described by Seki et al. [25,26]. At this point, lymphatic vessels are almost always present, just deeper into the superficial fascia. 

After the completion of fluorescence mapping, the marked areas were investigated by CDU both to confirm the presence and level of ectasic lymphatic vessels detected by fluorescence and to find venules suitable for LVA close to the target lymphatic vessel. Venules were considered suitable if similar in size and not involved by blood reflux during the Valsalva maneuver and compression of the skin downstream. Whenever possible, side branches of larger veins or venules’ bifurcation sites were chosen. The position of suitable venules was marked with a continuous black line on the skin. At the point of close proximity of a superficial reflux-free venule with the target lymphatic vessel, the site for LVA was marked. Finally, at the level of the groin, we investigated by CDU the position and depth of the functioning lymph-nodes detected at the preoperative lymphoscintigraphy, below the inguinal ligament in the Scarpa’s triangle, and planned the skin incision over them [7]. A neighboring vein, suitable for anastomosis, that preferentially was a branch of the great or accessory saphenous, superficial epigastric, or superficial circumflex iliac vein, was marked on the skin. LVA was performed by a previously described technique through intima-to-intima coaptation [30]. Whenever possible, only in the leg and in the thigh, both anterograde and retrograde flow were shunted by anastomosing both the proximal and distal stump of the lymphatic vessel to the venule, using end-to-end anastomosis in sites of bifurcation of the venule, or end-to-end anastomosis coupled with end-to-side anastomosis in the presence of single venular stump. In the latter case, the distal lymphatic stump, carrying the anterograde flow, was anastomosed in an end-to-end fashion, while the proximal stump, responsible for the retrograde flow, was connected end-to-side. After surgery, all patients underwent bandaging for two weeks and then were evaluated by the surgeon and the physical therapist for the prescription of a new compressive garment. The possibility of reduction in compression class or discontinuation of the garment was evaluated not before one year after surgery, according to the volume improvement, the absence of pitting, and to the softness of the limb.

### 2.2. Statistical Methods

The sample was described in its clinical and demographic features using descriptive statistics techniques. Quantitative variables were described using the following measures: minimum, maximum, range, mean, and standard deviation. Qualitative variables were summarized with absolute and percentage frequency tables. The normality of continuous variables was checked using the Shapiro–Wilk test. All values of pre and postoperative MC and volume were reported as the mean ± SD. The changes between preoperative and postoperative values of limbs’ measures were analyzed by Student’s *t*-test. *p* values < 0.05 were considered significant. All statistical analyses were performed with IBM^®^SPSS^®^Statistics software V.24 (IBM, Armonk, NY, USA).

## 3. Results

Between 2015 and 2021, 33 patients underwent LVA for the treatment of early-stage LEL after PL was performed for gynecological cancer. The patients’ age ranged from 33 to 78 years, with a mean of 56 years. BMI ranged from 18.3 to 38.3; mean of 24.5. Three patients were obese, and ten were overweight. Eighteen patients had received PL for cervical cancer, twelve for endometrial cancer, two for ovarian cancer, and one for vaginal/urethral cancer. The number of lymph nodes removed during the lymphadenectomy ranged from 8 to 52; mean of 28. In 29 patients, radiotherapy was associated with surgery. Ten patients were affected by hypertension, four by hypothyroidism, two by diabetes, one by epilepsy, one by emphysema, one by primary biliary cirrhosis, one by contracted kidney, and one by atrial fibrillation. In four patients, previous iliac venous thrombosis had occurred. The duration of swelling ranged from 10 months to 12 years; mean of 4 years. Previous lymphangitis was reported by 17 patients. Mean follow-up was 44.5 months. In every patient, three incision sites were employed to perform LVA. A total of 119 LVA were established with a mean of 3.6 (range 2–5). In 5 incision sites out of 99, the lymphatic vessel was found but not suitable for anastomosis due to the small size of the lumen and wall sclerosis. Lymphatic detection rate was 95% and vein detection rate was 100%.

**Figure 2:**
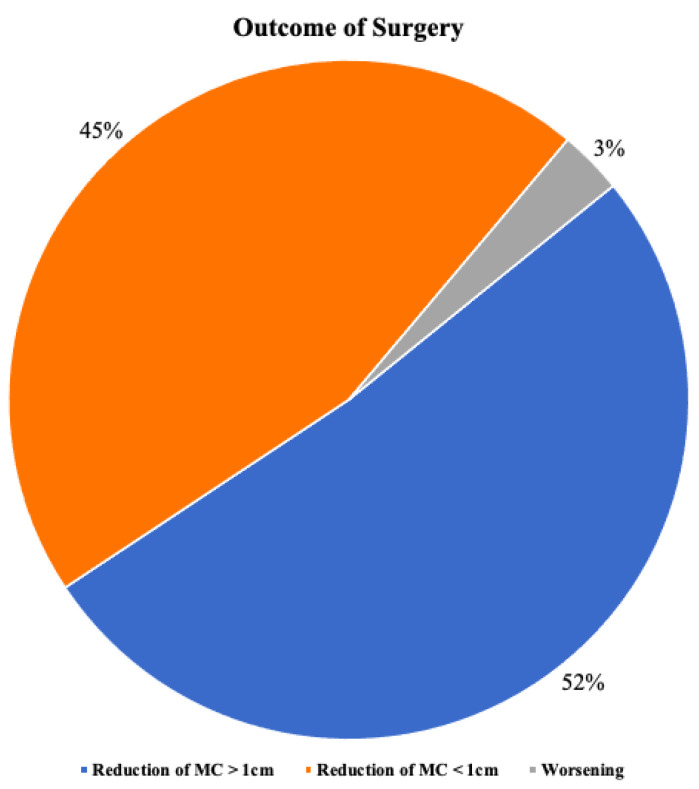
This pie graph shows the patients’ outcomes after surgery in terms of reduction in the mean circumference of the limb.

**Figure 3:**
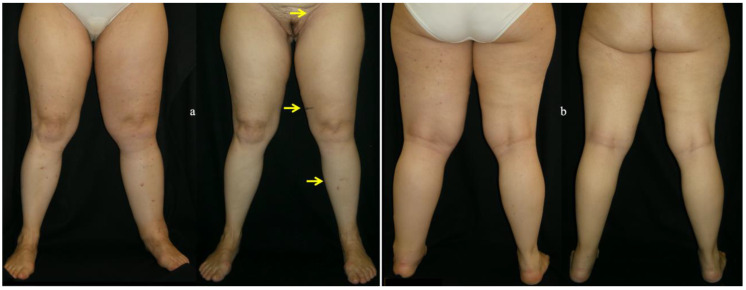
This figure shows anterior and posterior views of a patient affected by early-stage left LEL after PL for gynecological cancer, undergone Lympaticovenular Anastomosis (LVA), before and after surgery. (**a**) Anterior view of the patient before and after LVA was performed in the left lower limb. The yellow arrows indicate the sites where LVA was performed. After surgery, the improvement of swelling involves the whole limb; (**b**) posterior view of the same patient before and after surgery.

After surgery, in 17 patients (52%), the mean circumference of the operated limb decreased by more than 1 cm. In 15 patients (45%), the difference between pre and postoperative measures was less than 1 cm. One patient (3%) experienced worsening of swelling (Figure 2). This patient had a history of deep vein thrombosis after gynecological surgery. Considering all the included patients, the mean circumference of the operated limb showed a significant reduction after surgery (Figure 3), decreasing from 37 cm ± 4.1 cm to 36.1 cm ± 4.4 (*p* < 0.01).

## 4. Discussion

Secondary LEL is a chronic and progressive disease that negatively affects the QoL [32,33], causing psychological effects and physical symptoms, such as pain, numbness, and impaired mobility. Surgical treatment is indicated when conservative therapies are ineffective in maintaining a good decongestion. There is evidence in the literature that surgical treatment, combined with a correct physical therapy program, can achieve improvement that conservative treatments alone cannot provide. Such an improvement is particularly evident in some areas: function, frequency of infections, possibility to wear suits of the correct size, reduction in compression class of the garments, and frequency of physical treatments [34,35,36,37,38,39,40,41,42].

Among the available surgical techniques, supermicrosurgical LVA has gained widespread consensus worldwide due to its safety and efficacy in improving lymphedema symptoms, reducing swelling, attenuating disease progression, and decreasing the frequency of infections [43,44,45]. The anastomosis between lymphatic vessels and adjacent superficial venules allows redirecting lymph outflow to the venous system bypassing the obstruction and draining the excess fluids of the lymphedematous limb. Despite the fact that, in many centers, this procedure is the first-line option for lymphedema surgery, thanks to its minimal morbidity, the standard for a detailed procedure of LVA has not been established, including the number, location, and the pattern of anastomoses [25,46,47,48].

In its initial 2000 description, Koshima [49] recommended performing exploratory skin incisions for LVA in the lateral and medial aspects of the limb, assuming that lymphatic channels usually surround cutaneous veins. Nowadays, owing to the improvement of imaging techniques and to a better knowledge of the anatomy of the lymphatic system, this method is not employed in most centers because of its low probability of finding functional lymphatic vessels [50].

**Figure 4:**
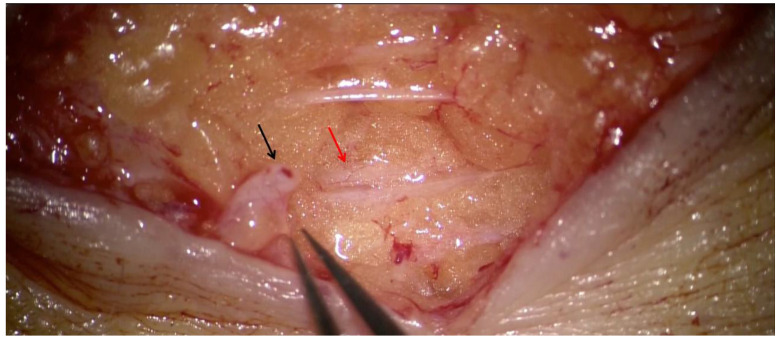
This figure shows the two important elements to achieve good outcomes after LVA. Red arrow indicates a lymphatic vessel with good size and wall that, during dissection, showed peristaltic movements; the black arrow indicates a reflux-free venule of similar size, suitable for anastomosis.

Many techniques are available to investigate the lymphatic system. Lymphoscintigraphy is the gold standard method for the diagnosis and first assessment of lymphedema but is not adequate for planning skin incisions because of its scarce spatial resolution. Recent studies have reported the increasing role of magnetic resonance, which can provide important anatomical and functional information, but its use to plan LVA is not standardized in many countries [50,51]. Among the available diagnostic instruments, mostly ICG-L and ultrasound have dramatically transformed the planning of LVA. In fact, the possibility of finding peristaltic and possibly ectasic lymphatic vessels with reflux-free venules (Figure 4) is currently considered by many authors the most important key factor in achieving good surgical outcomes after LVA [25]. 

The importance of the “quality” over the number of the anastomoses performed has been underlined by many authors [52]. In our opinion, the “quality” of every single anastomosis is determined by the size of the lumen and peristalsis of the lymphatic vessel, by the size match and absence of blood reflux of the venule, and by the position and distribution of the LVA throughout the limb, according to the areas of swelling. Dealing with the size of the lumen and peristaltic activity of the lymphatic vessel, in our opinion, ICG-L combined with ultrasound can provide the best information.

In early-stage patients, ICG-L allows understanding of the course, points of obstruction, and the presence of dilation of the lymphatic pathways. In our opinion, our technique of ICG injection allowed us to visualize the greatest possible number of lymphatic vessels because multiple injections at different levels throughout the limb minimizes the limitation caused by possible obstruction sites. Usually, in early-stage cases, the obstruction sites are few, but they result in DB that produces areas of diffuse fluorescence. This diffuse fluorescence can progress from distal to proximal, impairing the visibility of potential underlying functioning vessels located in sites proximal to the extravasation points. In the early phase of the exam, lymphatic vessels could be marked over the skin, following linear and splash patterns of fluorescence. The concept of “overlapping areas” on ICG-L, described by Yamamoto et al., is very important [28]. These points correspond to the transition between areas characterized by the prevalence of linear or ectasic patterns and areas dominated by DB patterns, especially stardust patterns. These passage areas are located immediately upstream, in relation to the lymphatic flow, to a site of obstruction that causes the reflux of fluorescence in the dermis (DB area); therefore, the lymphatic vessels that flow into these areas usually show a linear or splash pattern, are often dilated, and have increased peristalsis because they are immediately upstream to an obstruction site [9,13,53]. Dilated and peristaltic vessels are more suitable for LVA, can treat all the swollen area before the obstruction site, and have a better probability of anastomosis patency over time. Moreover, in our opinion, the position of the anastomosis immediately upstream of an obstruction site also has the advantage that, in the case of anastomosis thrombosis, a worsening of the swelling is unlikely. The evaluation with ultrasound after ICG-L has the double importance of confirming the position and the points of ectasia of the lymphatic vessels and detecting the venules of similar size and without blood reflux, possibly at the level of bifurcation or side branches [54,55,56]. Side branches are important for the frequent presence of valves nearby and because they allow anastomosing both the distal and proximal lymphatic stumps with end-to-end configuration, draining both the anterograde and retrograde lymphatic flow. In our opinion, this possibility is particularly important at the level of the so-called Seki point. In fact, this anastomosis has a strong therapeutic effect because the knee joint movement during walking works as a pump to propel lymph into the vein [25,26] and is in a central position in the limb, making the possibility of draining both the anterograde and retrograde flow important. Considering that LVA in most of the cases can improve lymph stasis in the area immediately distal to its location (proximal with respect to the lymph flow direction), our results suggest that, in early-stage cases following PL, the selection of the incision sites for LVA must be made whenever possible considering the three different anatomical subunits of the lower limb: leg; the lower third of the thigh; the upper third of the thigh. In fact, the LVA performed at the level of the upper leg is effective in mainly treating the swelling of the lower leg and of the foot; the LVA performed in the Seki point can improve the distal thigh and the upper leg and the one in the groin the middle and proximal part of the thigh. 

The main limitations of this study may be considered the absence of a control group treated using a different approach, the retrospective and single-center design. Nevertheless, the evaluation was carried out on a good number of patients, with a relatively long follow-up, and provided strong statistical significance. 

## 5. Conclusions

Supermicrosurgical LVA is a minimally invasive procedure that has gained popularity among lymphatic surgeons to treat secondary lower limb lymphedema thanks to its safety and efficacy and to the increasing efforts to improve and standardize patients’ selection and procedure planning and execution. Our results suggest that, in patients affected by early-stage LEL after PL for gynecological cancer, the placement of incision sites in all the anatomical subunits of the lower limb is one of the key factors in achieving good results. 

## Data Availability

The data presented in this study are available on request from the corresponding author.
